# Phylogeographic, genomic, and meropenem susceptibility analysis of *Burkholderia ubonensis*

**DOI:** 10.1371/journal.pntd.0005928

**Published:** 2017-09-14

**Authors:** Erin P. Price, Derek S. Sarovich, Jessica R. Webb, Carina M. Hall, Sierra A. Jaramillo, Jason W. Sahl, Mirjam Kaestli, Mark Mayo, Glenda Harrington, Anthony L. Baker, Lindsay C. Sidak-Loftis, Erik W. Settles, Madeline Lummis, James M. Schupp, John D. Gillece, Apichai Tuanyok, Jeffrey Warner, Joseph D. Busch, Paul Keim, Bart J. Currie, David M. Wagner

**Affiliations:** 1 Global and Tropical Health Division, Menzies School of Health Research, Darwin, Northern Territory, Australia; 2 Centre for Animal Health Innovation, Faculty of Science, Health, Education and Engineering, University of the Sunshine Coast, Sippy Downs, Queensland, Australia; 3 The Pathogen and Microbiome Institute, Northern Arizona University, Flagstaff, Arizona, United States of America; 4 Environmental and Public Health Microbiology Research Group, Microbiology and Immunology, James Cook University, Townsville, Queensland, Australia; 5 Tasmanian Institute of Agriculture, University of Tasmania, Hobart, Tasmania, Australia; 6 Translational Genomics Research Institute, Flagstaff, Arizona, United States of America; University of Texas Medical Branch, UNITED STATES

## Abstract

The bacterium *Burkholderia ubonensis* is commonly co-isolated from environmental specimens harbouring the melioidosis pathogen, *Burkholderia pseudomallei*. *B*. *ubonensis* has been reported in northern Australia and Thailand but not North America, suggesting similar geographic distribution to *B*. *pseudomallei*. Unlike most other *Burkholderia cepacia* complex (Bcc) species, *B*. *ubonensis* is considered non-pathogenic, although its virulence potential has not been tested. Antibiotic resistance in *B*. *ubonensis*, particularly towards drugs used to treat the most severe *B*. *pseudomallei* infections, has also been poorly characterised. This study examined the population biology of *B*. *ubonensis*, and includes the first reported isolates from the Caribbean. Phylogenomic analysis of 264 *B*. *ubonensis* genomes identified distinct clades that corresponded with geographic origin, similar to *B*. *pseudomallei*. A small proportion (4%) of strains lacked the 920kb chromosome III replicon, with discordance of presence/absence amongst genetically highly related strains, demonstrating that the third chromosome of *B*. *ubonensis*, like other Bcc species, probably encodes for a nonessential pC3 megaplasmid. Multilocus sequence typing using the *B*. *pseudomallei* scheme revealed that one-third of strains lack the “housekeeping” *narK* locus. In comparison, all strains could be genotyped using the Bcc scheme. Several strains possessed high-level meropenem resistance (≥32 μg/mL), a concern due to potential transmission of this phenotype to *B*. *pseudomallei*. *In silico* analysis uncovered a high degree of heterogeneity among the lipopolysaccharide O-antigen cluster loci, with at least 35 different variants identified. Finally, we show that Asian *B*. *ubonensis* isolate RF23-BP41 is avirulent in the BALB/c mouse model via a subcutaneous route of infection. Our results provide several new insights into the biology of this understudied species.

## Author summary

The pathogenic bacterium *Burkholderia pseudomallei* causes the disease melioidosis, which occurs in most tropical regions across the globe. The true burden of melioidosis is unknown but has been predicted to affect 165,000 people every year, resulting in 89,000 deaths. *B*. *pseudomallei* is easily confused with its close relative *B*. *ubonensis* as both species are frequently found in the same environmental niche and can appear phenotypically identical using serotyping and laboratory culture methods. *B*. *ubonensis* is a poorly characterised species but has recently gained interest in the research community as a potential biocontrol agent in *B*. *pseudomallei*-endemic regions, and for production of unusual and versatile biocompounds that are now being exploited for industrial applications. *B*. *ubonensis* is thought to be non-pathogenic, although other members of the *B*. *cepacia* complex to which it belongs are known for their ability to cause clinical disease that can be fatal in immunocompromised patients and people with cystic fibrosis. In this study, we investigated the biology of *B*. *ubonensis* to better understand its genetics, genomics, global distribution, virulence potential and antibiotic resistance. We show that this organism is highly genetically diverse, is avirulent in the mouse model, and can naturally encode high levels of meropenem resistance. We also identify *B*. *ubonensis* in the Caribbean for the first time, with phylogenomic analysis revealing distinct clades corresponding to geographic origin.

## Introduction

The Gram-negative soil- and water-dwelling bacterium *B*. *ubonensis* is a member of the *Burkholderia cepacia* complex (Bcc) [1], a genetically related group of metabolically diverse, highly adaptable and widely dispersed environmental species [2]. The Bcc, which comprises at least 20 species, includes some members known for their ability to cause clinical disease, such as severe sepsis in the immunocompromised and progressive pulmonary disease in cystic fibrosis patients [3]. Many Bcc species are also recognised for their unique biotechnological potential, particularly in bioremediation applications and in the production of antibiotic and antifungal compounds [4]. Novel compounds produced by *B*. *ubonensis* have been proposed as potential agents in biocontrol against *Burkholderia pseudomallei* [5] and in biodiesel catalysis [6].

*B*. *pseudomallei*, the causative agent of the tropical infectious disease melioidosis, is frequently isolated from the same soil samples as *B*. *ubonensis* in regions where both species are endemic [7]. Melioidosis is a diagnostically challenging and often deadly disease that affects humans and many animals, and remains underdiagnosed in many regions across the globe [8]. As *B*. *pseudomallei* is not a part of the healthy human flora, the ‘gold standard’ method for melioidosis confirmation is growth of *B*. *pseudomallei* from clinical specimens. For maximum isolation of *B*. *pseudomallei* from non-sterile sites such as sputum and pus, clinical laboratories require selective culture methods such as Ashdown’s agar containing gentamicin [9] and Ashdown’s broth containing colistin [10]. These media have also been used to successfully isolate *B*. *pseudomallei* from microbiologically complex environmental samples such as soil and surface water, which would otherwise yield growth and dominance of many other species [11]. We have previously demonstrated that *B*. *ubonensis* is the most commonly co-isolated species when using *B*. *pseudomallei* enrichment methods in the melioidosis-endemic “Top End” of the Northern Territory, Australia, in part due to the indistinguishable nature of certain *B*. *ubonensis* and *B*. *pseudomallei* morphotypes [7]. In addition, it has been reported that the atypical *B*. *pseudomallei* O-antigen type B is found in 25% of *B*. *ubonensis* strains from Australia [12], further complicating the differentiation between these species due to their immunological cross-reactivity.

Little is currently known about the population biology and genomics of *B*. *ubonensis*, although a clearer picture is emerging. The first *B*. *ubonensis* isolate (“*B*. *uboniae*” EY 3383, isolated from soil in Ubon Ratchathani in 1989) was reported in 2000 [13], and the first *B*. *ubonensis* genome (MSMB0022, isolated from soil in Darwin, Australia, in 2001) was sequenced to closure in 2015 [14]. The MSMB0022 genome encodes three circular replicons totalling ~7.2Mbp, which is approximately the same size as the two-chromosome *B*. *pseudomallei* genome. In Bcc species, the third replicon, a megaplasmid called pC3 (formerly chromosome III), has been shown to be important for stress resistance, virulence, and antifungal and proteolytic activity in several strains [15, 16]. This replicon is not essential for survival, with ~4% of tested Bcc isolates having spontaneously lost pC3, and additional strains able to be cured of this replicon either by plasmid incompatibility or by removal or toxin-antitoxin systems [15]. Although pC3 loss in *B*. *ubonensis* has been achieved *in vitro*, pC3 loss in wild-type *B*. *ubonensis* strains has not yet been identified.

Previous work has shown that Bcc species can encode for innate high-level resistance towards many clinically relevant antibiotics, including the carbapenem antibiotic meropenem [17]. Meropenem is a critical antibiotic for melioidosis therapy, being considered the treatment of choice for those with life-threatening sepsis [18, 19]. To date, the vast majority of *B*. *pseudomallei* isolates have been fully susceptible to meropenem [20], although recent evidence has shown that decreased susceptibility towards meropenem can occur after prolonged use of this antibiotic in melioidosis patients with severe sepsis [21]. Certain Bcc species such as *B*. *vietnamiensis*, *B*. *cepacia* and *B*. *cenocepacia* [22], as well as *B*. *pseudomallei* [23, 24], exhibit high rates of intra-species recombination. This observation raises the concern that antibiotic resistance genes may spread amongst *Burkholderia* species in the environment and potentially to the globally important pathogen *B*. *pseudomallei*.

The current study describes the first comprehensive analysis of the population biology of *B*. *ubonensis* from Australia and Asia. In addition, we identify the first *B*. *ubonensis* isolates from the Caribbean. Using large-scale comparative genome analysis, we interrogated 264 *B*. *ubonensis* genomes to better understand the geographic distribution and genetic diversity of this species, including potential loss of the pC3 megaplasmid. We also explored rates of meropenem resistance in Asian and Australian *B*. *ubonensis* strains, lipopolysaccharide (LPS) O-antigen cluster prevalence and diversity, and the virulence potential of an Asian *B*. *ubonensis* strain in the BALB/c mouse model.

## Methods

### Ethics statement

Procedures and ethics approval for collection of the environmental specimens from which the *B*. *ubonensis* isolates were recovered has been previously described [7, 25]. The murine challenge work was conducted according to the specific guidelines provided by the United States Department of Agriculture Animal Welfare Act under approved protocols from the Northern Arizona University IACUC (Protocol 14–011) and the USA Department of Defense Animal Care and Use Review Office (ACURO approval for HDTRA1-12-C-0066_Wagner).

### Isolates and species determination

The 264 *B*. *ubonensis* isolates examined in this study originated from northern Australia (*n* = 238), Central Australia (*n* = 4), Ubon Ratchathani, Thailand (*n* = 15), Papua New Guinea (PNG; *n* = 1), and Puerto Rico (*n* = 6), and were obtained from samples of soil (*n* = 160), water (*n* = 15), or plant material (*n* = 2) (S1 Table). DNA was extracted using protocols optimised for *B*. *pseudomallei* [26], and quality-checked using a NanoDrop UV spectrophotometer. Prior to WGS, all isolates were verified as *B*. *ubonensis* using the Bu550 real-time PCR [7], which targets the conserved iron-containing redox enzyme family protein encoded by *BW23_5472* on chromosome II of MSMB0022 (also referred to as MSMB22 [14]).

### Genome sequencing, assemblies and annotation

Genomic data were already publicly available for 230 of the 264 isolates [14, 27]. For completeness, we performed paired-end sequencing of the remaining 34 isolates using a HiSeq2000 instrument (Illumina Inc., San Diego, CA) at the Translational Genomics Research Institute (Phoenix and Flagstaff; AZ, USA). Assemblies were performed with the Microbial Genome Assembler Pipeline (MGAP; https://github.com/dsarov/MGAP---Microbial-Genome-Assembler-Pipeline), which incorporates Trimmomatic [28], Velvet [29], VelvetOptimiser (https://github.com/tseemann/VelvetOptimiser), GapFiller [30], PAGIT [31] and SSPACE [32] into its workflow, using the closed *B*. *ubonensis* MSMB0022 genome [14] as a reference for aligning, reordering and orientating contigs. All assemblies were quality-assessed by BLAST against phiX, with any contigs corresponding to this bacteriophage removed. Assemblies were annotated using PGAP [33]. Reference accessions for all 264 genomes are listed in S1 Table.

### Comparative genome analysis

The default settings of SPANDx v3.0 [34] were used to identify biallelic single-nucleotide polymorphisms (SNPs) from the 264 *B*. *ubonensis* genomes for phylogenetic analysis. *B*. *ubonensis* MSMB0022 was used as a reference genome for paired-end read alignment. BEDTools [35], which is run by default in SPANDx, was used to determine gene presence/absence relative to MSMB0022 using a 1kb locus ‘window’ size. Loci were considered variable if they had ≤99% read coverage in one or more strains, and conserved otherwise. To confirm the loss of pC3 (previously called chromosome III) in 10 isolates and to rule out alternative replicons being present in these strains, the unmapped reads from SPANDx for each strain were assembled using MGAP.

BEDTools was also used to determine LPS O-antigen type based on mapping quality against both known and novel LPS O-antigen clusters. Known clusters included *B*. *pseudomallei* K96243 (Type A LPS; GenBank reference BX571965.1; coordinates 3196645–3215231), *B*. *ubonensis* MSMB0057 (Type B LPS; GenBank reference JF745807), *B*. *pseudomallei* 576 (Type B LPS; GenBank reference NZ_CP008777.1; coordinates 1383179–1418799), *B*. *ubonensis* MSMB0122 (Type B2 LPS; GenBank reference HQ908420.1), *B*. *pseudomallei* MSHR0840 (Type B2 LPS; GenBank reference GU574442.1), *B*. *thailandensis* 82172 (Type B2 LPS; GenBank reference JQ783347.1) and *B*. *humptydooensis* MSMB0043 (novel LPS; GenBank reference CP013380.1; coordinates 971381–996024). *B*. *ubonensis* type strains for determining the prevalence of novel LPS O-antigen genotypes were: A21, BDU9, BDU12, BDU14, INT1-BP158, MSMB0022, MSMB0054, MSMB0063, MSMB0083, MSMB0103, MSMB0268a, MSMB0609, MSMB0742, MSMB0782, MSMB0827, MSMB1058, MSMB1137, MSMB1172, MSMB1173, MSMB1178, MSMB1189, MSMB1206, MSMB1304, MSMB1471, MSMB1517, MSMB1586, MSMB1591, MSMB2105, MSMB2123, MSMB2166, MSMB2180, MSMB2207, RF23-BP17, and RF32-BP11. Sequence coordinates for these LPS O-antigen clusters were extracted from MGAP-assembled genomes based on Mega BLAST analysis against the MSMB0057 O-antigen biosynthesis cluster [12].

### Multilocus sequence typing (MLST)

*In silico* MLST was carried out on all isolates using the Bcc scheme (http://pubmlst.org/bcc/), and on 173 of the 264 *B*. *ubonensis* isolates based on the *B*. *pseudomallei* scheme (http://pubmlst.org/bpseudomallei/). Ninety-one strains could not be genotyped using the *B*. *pseudomallei* scheme as they lack the *narK* housekeeping locus [36]. Sequence types (STs) were determined from assemblies using the BIGSdb tool, which is integrated into these MLST websites [37]. ST assignments for both schemes are listed in S1 Table and are also searchable on the online databases.

### Phylogenetic analysis

The maximum parsimony function of PAUP v4.0a153 [38] was used for phylogenetic reconstruction of genome-wide variants. The Ortho_SNP_matrix.nex output automatically generated by SPANDx was used as the PAUP input. Trees were constructed based on a heuristic search and bootstrapped using 100 replicates. FigTree (http://tree.bio.ed.ac.uk/software/figtree/) was used to visualise PAUP outputs.

### Laboratory passaging for pC3 megaplasmid loss

To promote pC3 loss *in vitro*, phylogenetically unrelated *B*. *ubonensis* strains MSMB0782 and MSMB1215 were passaged five times on Ashdown’s agar (37°C for 24-48h), and strains INT1-BP274 and RF23-BP41 were passaged 10 times. MSMB0782 and INT1-BP274 were also subjected to five freeze/thaws ranging from -80°C to room temperature, and INT1-BP274 was passaged seven times at 42°C or room temperature. Eighteen colonies of MSMB2036, which is the same ST as the pC3-negative strain MSMB2035, were then examined for pC3 loss by passaging once on Luria-Bertani agar and growing at 37°C for 48h. DNA from all laboratory-passaged strains was extracted using a chelex heat soak procedure [39] and diluted 1:10 prior to PCR. pC3 detection was carried out with primers Bu_pC3_For1 (5’-CGATGAGCTATTCGTTCGATCT) and Bu_ pC3_Rev1 (5’-AACGTGATCCGGTACAGCAC) to generate a 52bp amplicon, using a slowdown PCR for GC-rich templates [40]. MSMB2035 was included as the pC3-negative control. All DNA was verified for quality using the Bu550 assay [7].

### Minimum inhibitory concentration (MIC) determination

Etests (bioMérieux, Baulkham Hills, NSW, Australia) were used to determine meropenem MICs in 40 *B*. *ubonensis* strains (S1 Table). This subset of strains was chosen to represent geographically and phylogenetically diverse taxa, and to identify potential MIC differences among strains of the same ST. Isolates were grown on Mueller Hinton agar for 24h at 37°C in an oxygenated environment prior to MIC assessment.

### *B*. *ubonensis* murine challenge

The ability of *B*. *ubonensis* to cause disease via the subcutaneous (sc) route of infection was examined in a murine BALB/c model using a Thai environmental isolate, RF23-BP41 (S1 Table), collected by Northern Arizona University in 2007. We compared the results to sc infection with *B*. *thailandensis* type strain E264, which is known to cause death in mice at high doses (>10^6^ colony forming units, or CFU) when delivered via the intraperitoneal [41], intranasal [42, 43] or aerosol [44] routes. Virulence testing was performed in a similar manner as previously described [45]. After shipping, mice were acclimatised for five days before the experiment; food and water were provided *ad libitum* throughout the study. Mice were lightly anaesthetised with vaporised isoflurane and injected via a single 100μL sc injection in the scruff of the neck. All mice in a single cage received the same infectious dose (*B*. *ubonensis*: 1.71 x 10^4^, 10^5^ or 10^6^ CFU). Three infection control mice were injected in an identical way, but with 100μL of sterile 1x PBS instead of bacterial culture. Mice were monitored daily for health status and euthanased on day 21 post-injection with CO_2_ gas followed by exsanguination.

## Results and discussion

### Phylogeographic analysis of *B*. *ubonensis*

The true global distribution of *B*. *ubonensis* is not known. To date, strains have only been reported from the environment in Wuhan, China [6], Ubon Ratchathani, Thailand [13], northern and Central Australia [7], and PNG [5]. In this study, we identified *B*. *ubonensis* in the Caribbean environment for the first time, with six isolates retrieved from soil obtained from the north-central and north-eastern regions of Puerto Rico (Juncos, Ceiba and Barceloneta). A recent study of soil samples in the southern United States to determine the presence of *Burkholderia* spp., and particularly *B*. *pseudomallei*, did not yield a single *B*. *ubonensis* or *B*. *pseudomallei* isolate, although several other Bcc species were retrieved [40]. It is thus probable that neither *B*. *ubonensis* nor *B*. *pseudomallei* are naturally found in the environment in North America. It remains to be determined whether *B*. *ubonensis* is found in other melioidosis-endemic regions such as Africa, Central America, the Indian Ocean islands, South America or South Asia.

**Fig 1:**
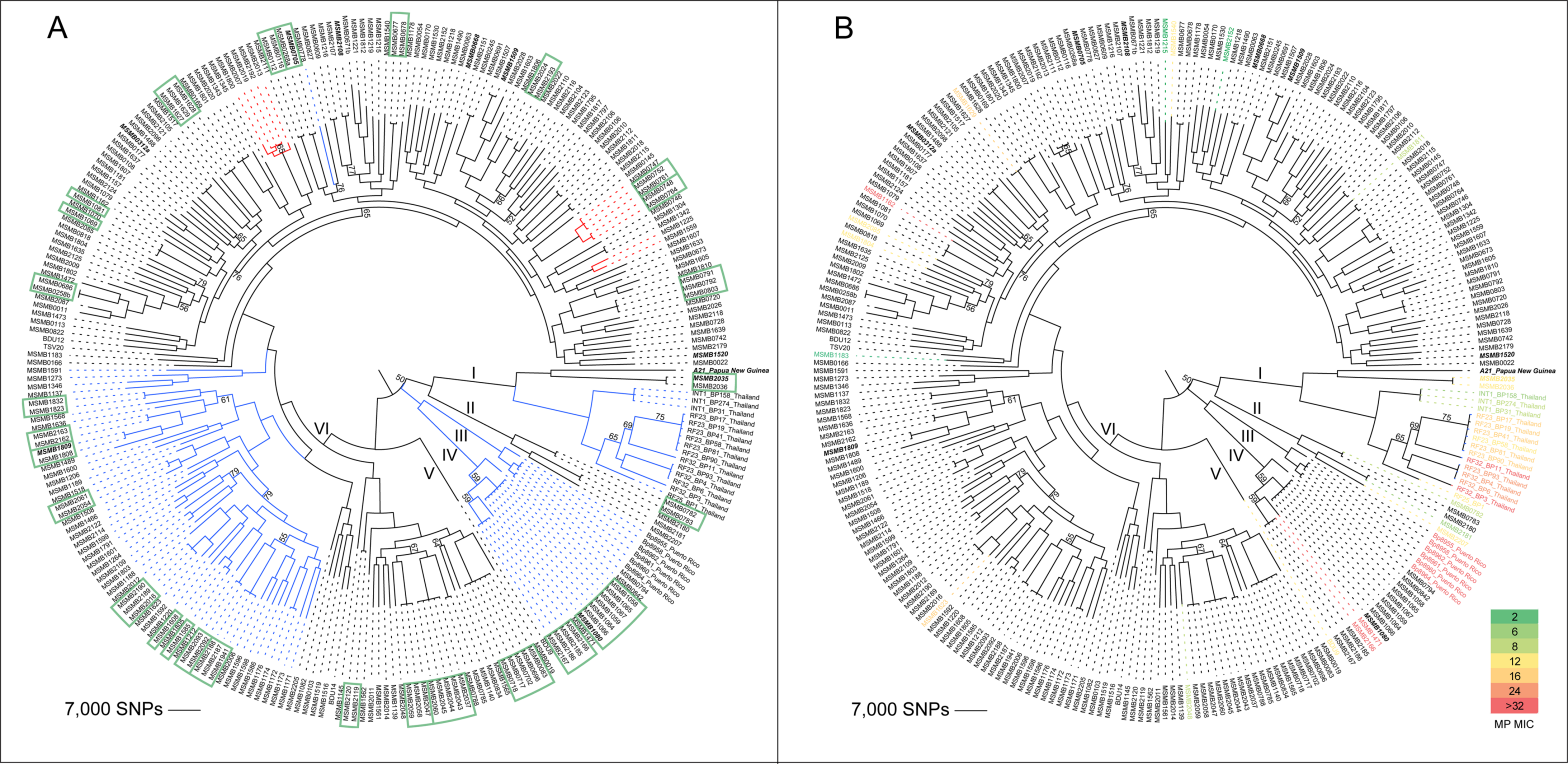
Phylogenomic analysis of 264 *Burkholderia ubonensis* genomes. A midpoint-rooted maximum parsimony phylogeny was constructed using 589,433 biallelic core-genome SNPs. A) Strains lacking the *B*. *pseudomallei* multilocus sequence typing (MLST) gene *narK* are labelled with blue branches, and those lacking pC3 (previously known as chromosome III) are in bold italics. Highly related strains retrieved from single environmental samples are outlined by green boxes. Red branches indicate instances where isolates could be differentiated by the *B*. *pseudomallei* MLST scheme, but not the Bcc scheme. B) Heatmap of the meropenem minimum inhibitory concentration values for 40 tested *B*. *ubonensis* isolates. In both trees, the six distinct clades (I, II, III, IV, V and VI) are labelled. Consistency index = 0.25. Bootstrap values below 80% are labelled on their corresponding branches.

A *B*. *ubonensis* phylogeny was reconstructed from 264 genomes derived from Australian, Thai, PNG and Puerto Rican isolates to determine the existence of a continental phylogeographic signal, a phenomenon that has been described in *B*. *pseudomallei* [23, 46, 47]. Based on 589,433 biallelic SNPs, six distinct and well-supported clades were identified. Clades II, IV, V and VI solely contained Australian *B*. *ubonensis* isolates (*n* = 240), whereas Clade I contained all isolates from Thailand (*n* = 15), the PNG isolate A21, and two Australian strains from the tropical “Top End” region of the Northern Territory, and Clade III was comprised of the six Puerto Rican isolates (Fig 1; S1 Fig). Subclades within Clade I showed that the Thai strains clustered most closely with one another (Fig 1), with A21 residing on its own branch and the two Australian strains, MSMB2035 and MSMB2036, sharing a node with the PNG isolate. The Puerto Rican isolates share a node with the Clade IV Australian isolates (Fig 1). Due to limited availability of *B*. *ubonensis* from PNG, it could not be determined whether other PNG isolates group with A21, although we hypothesise that PNG *B*. *ubonensis* strains will be related based on the relatively narrow genetic diversity observed in PNG *B*. *pseudomallei* populations [47, 48]. Within Clade IV, four isolates from the arid region of Central Australia (MSMB2166, MSMB2167, MSMB2185 and MSMB2186), which were obtained from the same soil sample, grouped with other Australian strains, with the most closely related isolates originating from the “Top End” region. Taken together, these results demonstrate that, like *B*. *pseudomallei*, *B*. *ubonensis* populations exhibit a continental phylogeographic signal, although more samples from Asia and PNG would be needed to improve resolution of subclades within Clade I.

### Comparison of phylogenomic structure and MLST

We compared *B*. *ubonensis* MLST genotypes obtained using both the *B*. *pseudomallei* and Bcc MLST schemes with phylogenomic assignment to determine whether the STs reflected isolate relatedness on the genome level [49], or whether homoplasy was evident among STs as has been observed with certain *B*. *pseudomallei* STs [50, 51]. For both MLST schemes, the ST and genomic data showed excellent concordance and no evidence of ST homoplasy, with all identical STs clustering closely on the phylogeny (Fig 1A; green box outlines) and non-identical STs residing on separate branches. Unlike the Bcc scheme, where STs could be assigned from all genomes, STs were not able to be determined for 91 (35%) isolates using the *B*. *pseudomallei* MLST scheme due to these strains lacking the “housekeeping” locus *narK* [36]. We identified five separate clusters within our phylogeny that lacked *narK* (Fig 1A; blue branches). The first included all the Thai isolates (*n* = 15), with the remaining four comprising all Puerto Rican (Clade III; *n* = 6) and Clade IV (*n =* 14) isolates, plus 57 isolates within Clade VI that were isolated from various “Top End” locales. These results show that certain *B*. *ubonensis* strains cannot be fully genotyped with the *B*. *pseudomallei* MLST scheme. However, in three instances where strains could be genotyped, the *B*. *pseudomallei* scheme was superior at differentiating strains that were related yet distinct on a genomic level (Fig 1, red branches; S1 Table). MSMBs 1225 and 1559 were both ST-1187 using the Bcc scheme but were different STs using the *B*. *pseudomallei* scheme; MSMB2013 was assigned ST-1235 by both schemes but the other Bcc ST-1235 strains were found to be ST-1226 according to the *B*. *pseudomallei* scheme; and the Bcc ST-1148 strains were separated into ST-1266 and ST-1267 based on the *B*. *pseudomallei* alleles. In all cases where additional STs were found, the isolates were obtained from distinct soil samples, indicating greater resolving power of the *B*. *pseudomallei* MLST scheme in these cases.

### Comparison of phylogenomic structure and meropenem MICs

**Fig 2:**
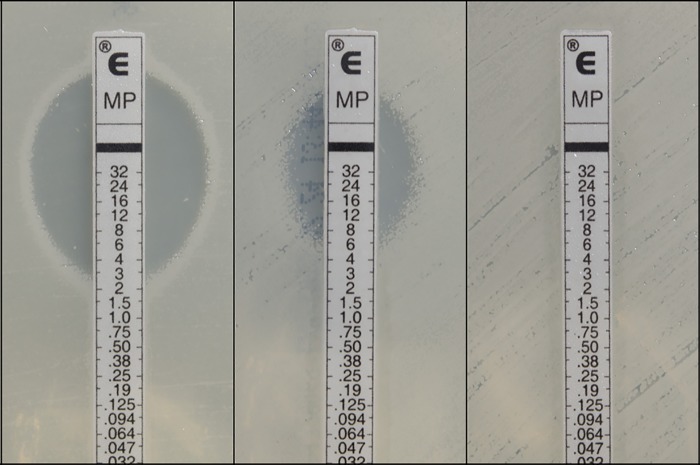
Example Etest results in *Burkholderia ubonensis* towards meropenem. Left, sensitive isolate MSMB2152 at a minimum inhibitory concentration (MIC) of 3 μg/mL; centre, intermediate isolate MSMB1183 at an MIC of 6 μg/mL; right, resistant isolate MSMB1162 at an MIC of ≥32 μg/mL.

We mapped meropenem MICs for 40 strains against the genome phylogeny to ascertain whether meropenem-resistant, meropenem-intermediate or meropenem-sensitive strains belonged to a single clade. Eleven strains (Bp8955, Bp8958, Bp8960, Bp8961, Bp8962, Bp8964, MSMB1162, MSMB1471, MSMB2166, RF32-BP11 and RF32-BP3) showed high-level resistance (≥32 μg/mL) towards this antibiotic, including all six Puerto Rican strains. In contrast, two Australian strains (MSMB1215 and MSMB2152) exhibited the lowest MICs at 2–3 μg/mL (Fig 2). Both highly resistant and highly sensitive (2–6 μg/mL) strains were found in the Asian and Australian populations, demonstrating that these phenotypes are not restricted to a certain clade and that *B*. *ubonensis* populations from these two geographic regions encode for a range of meropenem MICs (Fig 1B). Although our testing was not comprehensive, we did observe similar MICs for closely related strains. For example, the closely related Thai strains RF23-BP93, RF32-BP4 and RF32-BP6 all exhibited MICs of 24 μg/mL (Fig 1B). The lack of phylogenetic congruence of high-level meropenem-resistant strains supports the hypothesis that the genetic mechanism conferring resistance is laterally transferred among strains. Alternatively, resistance may have arisen multiple times or through multiple mechanisms during the evolution of *B*. *ubonensis* due to similar environmental pressures.

Many other Bcc species strains can exhibit high-level meropenem resistance [17, 52], indicating that this trait is not specific to *B*. *ubonensis*, although the basis for this resistance and its persistence in Bcc populations is not clear. In comparison, the highest meropenem MICs recorded for *B*. *pseudomallei* to date are ~4 μg/mL [53, 54], with wild-type strains consistently exhibiting MICs of 0.75–1 μg/mL. Unlike *B*. *pseudomallei*, where human-to-human transmission is exceptionally rare and where infections are almost always acquired from the environment [55], Bcc species can transmit between individuals, and indeed this a major clinical issue in the management of cystic fibrosis cohorts [56]. The selective forces acting upon Bcc strains in patients receiving meropenem or other antibiotics may encourage this phenotype to persist in the population, although the lack of human *B*. *ubonensis* infections and the identification of high-level meropenem resistance in environmental samples argue against this route of selection in the context of *B*. *ubonensis*. *B*. *pseudomallei* does not encode a carbapenamase, which likely explains why high-level resistance has not been reported. However, it is conceivable that *B*. *pseudomallei* may acquire a carbapenamase whilst residing in the environment, especially from closely related species that share this niche, such as *B*. *ubonensis* or other Bcc species. Determining the molecular basis for high-level meropenem resistance in *B*. *ubonensis* and in other Bcc species should be a focus of future studies to not only promote a better understanding of resistance mechanisms in these species, but to also provide a basis for proactive monitoring of *B*. *pseudomallei* populations in the event of carbapenamase acquisition.

### Genetic diversity of *B*. *ubonensis*

MLST revealed that *B*. *ubonensis* is a highly diverse species. We found 128 STs among the 173 strains that could be genotyped using the *B*. *pseudomallei* MLST scheme, and 182 STs among the 264 strains based on the Bcc scheme, although these numbers underestimate diversity due to multiple related isolates being tested from single environmental specimens in our study (S1 Table). Among the 33 Bcc scheme STs represented by two or more *B*. *ubonensis* isolates, 27 (82%) of these STs were found within a single sample; such samples are likely to be identical or clonally related due to their physical proximity. We next examined *B*. *ubonensis* diversity within our environmental samples. Of the 51 samples where two or more *B*. *ubonensis* isolates were retrieved, 26 (51%) exhibited two or more STs, revealing that multiple *B*. *ubonensis* genotypes commonly exist within single environmental samples. This result reflects similar observations made in studies examining *B*. *pseudomallei* diversity in environmental samples from Thailand [57, 58], *B*. *vietnamiensis* in the United States [40], and *B*. *cepacia* genomovar III (now known as *B*. *cenocepacia*) in the United States, Canada and Australia [59]. Whilst isolation of multiple colonies from a single sample is a laborious endeavour, these studies reinforce the need to collect multiple isolates from individual samples to maximise capture of population diversity.

### The megaplasmid pC3 is nonessential to *B*. *ubonensis* replication

**Fig 3:**
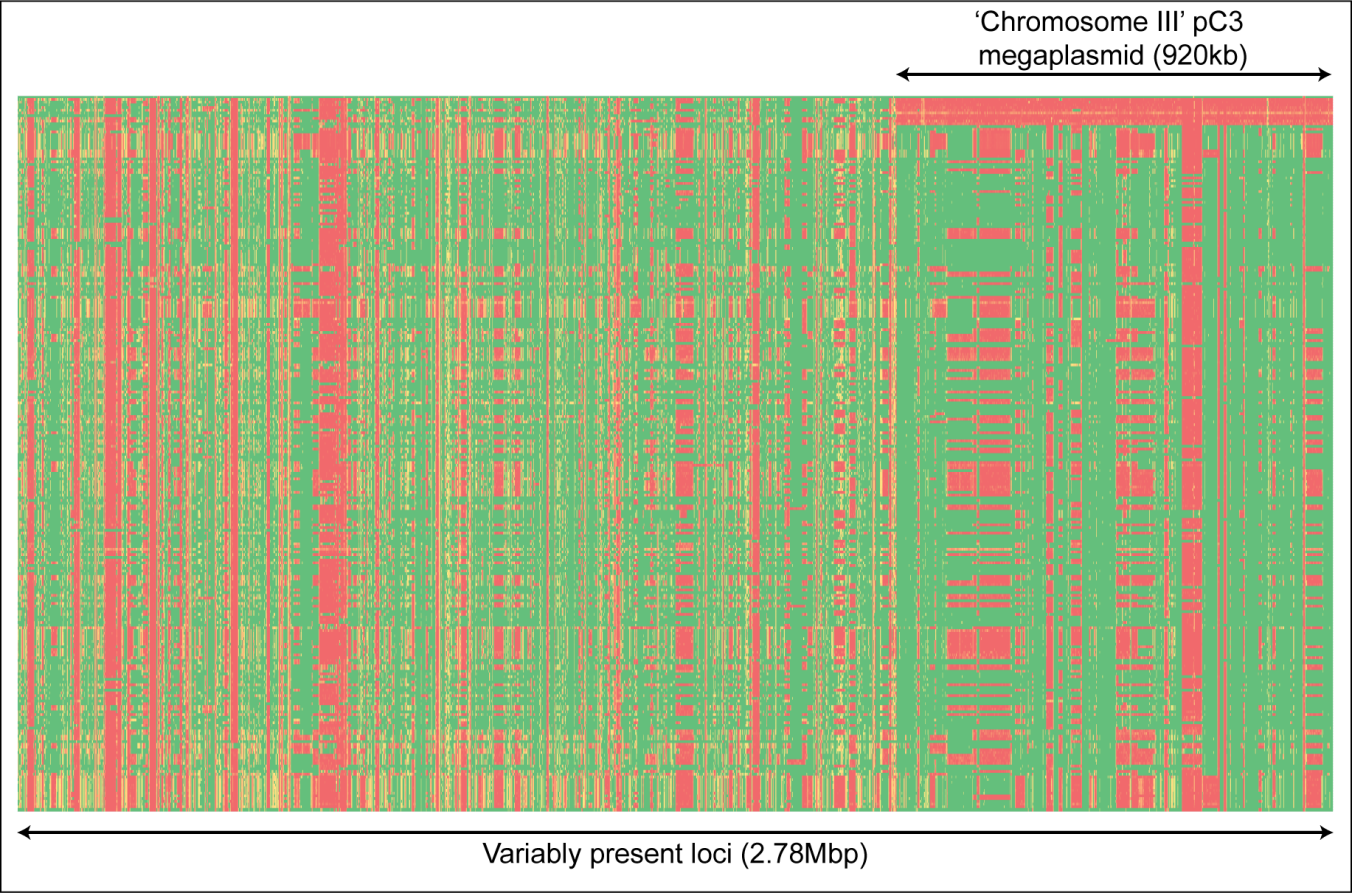
Heatmap of variably present *Burkholderia ubonensis* genes across the MSMB0022 genome. A presence/absence matrix was constructed across 1kb windows of the MSMB0022 reference genome for each of the 264 taxa. Green = 100% mapped reads; red = 0% mapped reads. Taxa are in rows and the 1kb windows are in columns. Only regions containing <80% window coverage for at least one strain are shown, representing 2.78Mbp of the MSMB0022 *B*. *ubonensis* genome. The absence of pC3 in 4% of strains demonstrates that this megaplasmid can occasionally segregate, a finding consistent with pC3 in other Bcc species [15].

Gene presence/absence analysis of the 264 *B*. *ubonensis* genomes against the MSMB0022 reference showed that 2.78Mbp (39%) of the *B*. *ubonensis* reference genome was variably present, with the remaining 4.41Mbp conserved across these strains. Ten phylogenetically unrelated strains (A21, MSMB0312a, MSMB0668, MSMB0705, MSMB1080, MSMB1509, MSMB1520, MSMB1809, MSMB2035 and MSMB2108) failed to map reads against the entire sequence for pC3, equating to one-third of the variable regions observed in our dataset (Fig 3). Certain closely related strains did not share this pattern: for example, MSMB2035 and MSMB2036 are clonal according to the two MLST schemes and the WGS phylogeny, yet only MSMB2035 lacked this replicon. Phylogenetic reconstruction using just pC3 as the reference showed no evidence of lateral transfer, with the topology of the tree being highly similar to the phylogenetic tree constructed for chromosomes I and II (Fig 1). This result suggests that pC3 is probably ubiquitous in *B*. *ubonensis* strains found in the environment and that it largely follows a vertical path of evolution, but, when propagated under certain conditions, segregation of this replicon can occur spontaneously; in our study, segregation occurred in 4% of strains. Agnoli and coworkers (2014) also observed that four of 110 Bcc isolates tested in their study (4%) had lost pC3, with one of these events having been confirmed to have occurred following laboratory passage [15]. In the type strain MSMB0022, pC3 encodes for 669 genes that are involved in myriad functions (S2 Table). When excluding this replicon, 1.86Mbp (26%) of the *B*. *ubonensis* reference genome was variable among the 264 strains.

The conservation of pC3 and its phylogenetic relatedness to chromosomes I and II confirms that pC3 is under strong selection pressure to be maintained in Bcc species, including *B*. *ubonensis*. However, certain growth conditions appear to encourage pC3 segregation, raising the possibility that this replicon may be a megaplasmid [60]. Based on the earlier work of Agnoli and colleagues [15, 16], we attempted to cure *B*. *ubonensis* strains MSMB0782, MSMB1215, INT1-BP274 and RF23-BP41 of pC3 by performing laboratory passage and growth under varying conditions, including multiple freeze/thaws, growth at 42°C and room temperature, or multiple passages. Despite these attempts, none were successful at segregating pC3. To examine whether an insufficient number of colonies were being tested, we next attempted passage of 18 colonies of MSMB2036, which is closely related to the pC3-lacking strain MSMB2035. Four (22%) colonies lost pC3 after a single passage on Luria-Bertani agar at 37°C for 48h, as observed by a lack of amplification using the Bu_pC3 primers. This finding demonstrates that, as with other Bcc species, the third replicon of *B*. *ubonensis* is not necessary for the organism’s survival, at least in a laboratory setting. It remains to be determined whether pC3 replicates independently of the two chromosomes in *B*. *ubonensis*. It has been proposed that the second (and where applicable) third ‘chromosomes’ found in approximately 10% of bacterial genomes are in fact ‘chromids’, a term used to define replicons that are not strictly chromosomes or plasmids [61]. To maintain consistency with the work of Agnoli and colleagues [15, 16], we have chosen to refer to this replicon as a pC3 megaplasmid.

At 920kb, the *B*. *ubonensis* pC3 megaplasmid is unusually large, although such size is not unprecedented, with *B*. *cenocepacia* H111 encoding a curable 1.04Mbp pC3 megaplasmid [16]. Larger megaplasmids have been identified in other soil- and rhizosphere-dwelling organisms including a 1.8Mbp linear megaplasmid identified in the actinomycete *Streptomyces clavuligerus* [62], and a 1.59Mbp megaplasmid in *Azospirillum brasilense* [63]. The pC3 replicon of *B*. *ubonensis* MSMB0022 failed to be detected as a plasmid using the online PlasmidFinder and VecScreen tools; however, we found that these tools also failed to identify the *B*. *vietnamiensis* megaplasmid pBVIE01, possibly because PlasmidFinder has been optimised for plasmid identification in Enterobacteriaceae [64]. BLAST analysis of *parA* and *parB* genes from *B*. *vietnamiensis* G4 pBVIE01 showed weak evidence of these partitioning system genes in MSMB0022 pC3, although more solid BLAST hits were obtained with chromosome I genes. This result does not rule out the presence of plasmid maintenance loci encoded on this replicon, but rather demonstrates the difficulties in identifying genetic homology across distantly related species. Similarly, the presence of 5S, 16S and 23S ribosomal RNA-encoding genes on pC3 does not necessarily rule out this replicon as being a megaplasmid [16, 60]. Read depth coverage analysis of pC3 showed similar depth to the two chromosomes (e.g. MSMB0011: 108x for pC3 vs 123x for chromosome I and 124x for chromosome II), indicating that this megaplasmid is at a low or single copy number, a finding that is consistent with the generally low copy number of larger plasmids [65].

### *B*. *ubonensis* exhibits high levels of LPS O-antigen diversity

Earlier work has shown that 25% of Australian *B*. *ubonensis* strains possess the unusual *B*. *pseudomallei* type B LPS O-antigen [12]. Using our larger dataset, we examined LPS diversity among the 264 strains *in silico*. Due to insufficient contig coverage across the LPS cluster, 19 strains could not be fully genotyped using this approach; however, these strains did not possess clusters matching to other LPS types. Of the remaining 245 strains that could be genotyped, type B LPS was identified in 20 (8%). In total, 35 different LPS types were found, compared with only four LPS types among 477 global *B*. *pseudomallei* strains using the same *in silico* approach. The most abundant LPS type in the *B*. *ubonensis* cohort was MSMB0063 Type Novel, with 28 strains having this genotype; in contrast, eleven LPS types were seen in only a single isolate (S1 Table). LPS genotypes were not restricted to particular STs or geographic regions. For example, the Thai strains RF25-BP1 and RF32-BP3 possessed an LPS cluster that was also found in Australian strains MSMB0782, MSMB0783, MSMB1188, MSMB1562, MSMB1603, and MSMB1635, and among these eight isolates, seven different STs were present. Our findings are consistent with the presence of similar LPS types among *Burkholderia* species. In addition, we show that *B*. *ubonensis* LPS is highly variable and is not associated with the genetic relatedness or geographic origin of an isolate, and would thus be a poor marker for such purposes.

### *B*. *ubonensis* RF23-BP41 does not cause disease in the immunocompetent BALB/c mouse model

Unlike other Bcc species or *B*. *pseudomallei*, *B*. *ubonensis* is thought to rarely, if ever, cause disease in humans [66], as evidenced by *B*. *ubonensis* being the only Bcc species not yet retrieved from cystic fibrosis sputum [52]. Indeed, there is only a single report of *B*. *ubonensis* being isolated from a human infection, a Thai nosocomial case (strain LMG 24263 [1]). Given the absence of other reported *B*. *ubonensis* infections to date, the role of *B*. *ubonensis* as the aetiologic agent in this Thai case should be treated with scepticism; for instance, testing for the presence of known pathogens in the same clinical specimen was not stated. However, another possibility is that certain *B*. *ubonensis* strains are in fact capable of causing disease, with such cases remaining unreported due to insufficient or inaccurate differentiation of *B*. *ubonensis* from other Bcc species.

To further examine the virulence potential of *B*. *ubonensis*, we inoculated BALB/c mice via sc injection using 1.7x 10^4^, 10^5^, and 10^6^ CFU of the Thai strain RF23-BP41. To our knowledge, *B*. *ubonensis* virulence has not yet been tested in the mouse model. RF23-BP41 was chosen for several reasons. First, its Thai origin maximises the probability of genetic relatedness to the putatively pathogenic LMG 24263 strain. Second, RF23-BP41 was isolated from a region where individuals (particularly rice farmers) regularly come into contact with soil bacteria, increasing the likelihood of successful human infection. Third, this strain demonstrated resistance towards meropenem (MIC 16μg/mL), which would potentially confer a selective advantage during antibiotic treatment. Finally, this strain harbours pC3, which has been shown to impart virulence capacity in other Bcc species [15, 16]. Even at the highest dose of 1.7x10^6^ CFU, no mice exhibited weight loss or lethargy during the 21-day challenge experiment, with their health status identical to that of the three control mice. The same result was observed in the BALB/c mice subcutaneously injected with *B*. *thailandensis* E264 at a similar dosage range [45]. Certain *B*. *thailandensis* strains are capable of infecting immunocompromised humans [67–69], and can be lethal in murine models when administered at high doses via other routes [41–44]. In contrast, in other studies the 10-day LD_50_ of *B*. *pseudomallei* in BALB/c mice was ~1x10^3^ CFU when delivered via the sc route [70], and between 10 and 6x10^4^ CFU when administered via the intraperitoneal route, with virulence reduced but not abolished in highly laboratory-passaged strains [71, 72]. Other mouse model studies have shown that virulence of Bcc species can vary; for example, the epidemic *B*. *cenocepacia* strain J2315 caused universal mortality when inoculated at 10^3^ cfu into gp91^phox−/−^ mice via an intratracheal route, whereas other *B*. *cenocepacia* strains were less virulent and *B*. *vietnamiensis* strain R2 was avirulent [73]. Another study using intranasal inoculation of leukopaenic BALB/c mice with ~10^4^ cfu also showed differential virulence within Bcc species, with some mice clearing their infections [74], indicating that virulence potential varies among strains.

Based on the findings of these earlier studies, pathogenicity may also vary among *B*. *ubonensis* strains. Characterising the virulence potential of other *B*. *ubonensis* strains may identify unusual pathogenic strains, although we deem this unlikely based on the lack of verified human infections caused by *B*. *ubonensis*. In consideration of the IACUC guidelines, we chose not to carry out testing of further strains using the mouse model. We acknowledge that our study only tested *B*. *ubonensis* in immunocompetent BALB/c mice via a sc route. The use of immunocompromised or immune-deficient mouse models or infection via different routes may reveal that *B*. *ubonensis* can cause disease in such cases. Bcc species carry various virulence factors that are thought to contribute to their pathogenic potential, including extracellular lipases, metalloproteases, serine proteases, flagella, pili, adhesins, toxins, siderophores and lipopolysaccharides [75]. We did not investigate the presence of virulence genes in *B*. *ubonensis* compared with other Bcc species but doing so may shed further light on its potential virulence capacity. It may be possible to use such *in silico* methods rather than further animal experiments to determine whether *B*. *ubonensis* is unusual compared with other Bcc species due to a lack of key virulence loci or pathways in its genome.

## Conclusions

The metabolic diversity of Bcc species continues to spur interest in this highly adaptable group of bacteria. Our study provides important new insights into the biology of *B*. *ubonensis*, a largely neglected member of the Bcc due to its ostensibly avirulent nature. Genomic analysis of 264 *B*. *ubonensis* strains from Australia, PNG, Puerto Rico and Thailand revealed that *B*. *ubonensis* is a genetically highly diverse organism, with at least 26% of its chromosomal DNA variably present among strains. Like *B*. *pseudomallei*, *B*. *ubonensis* has a distinct phylogeographic signature that can be distinguished at the genomic level. It remains to be determined whether *B*. *ubonensis* is found on other continents. ‘Chromosome III’ encodes a ubiquitous yet apparently dispensable pC3 megaplasmid, similarly to other Bcc species, and can segregate in the laboratory setting. Like other Bcc species, we show that *B*. *ubonensis* strains exhibit variable levels of meropenem resistance. Determining the molecular mechanism underpinning high-level meropenem resistance in certain *B*. *ubonensis* strains will provide a better understanding of the potential transmission of this phenotype to the melioidosis bacterium *B*. *pseudomallei*, which frequently co-resides with *B*. *ubonensis* in the environment. Finally, using the immunocompetent BALB/c mouse model, we show that an Asian *B*. *ubonensis* strain is not likely to cause disease, providing evidence that at least some members of this species are probably avirulent in immunocompetent individuals. Further studies are needed to confirm the avirulent nature of *B*. *ubonensis* across a greater strain set using both immunocompetent and immunocompromised or immunodeficient animal models, or *in silico* analysis of the *B*. *ubonensis* genome to identify intact virulence determinants. The apparent non-pathogenic nature of certain *B*. *ubonensis* strains may make them amenable to large-scale biotechnological applications, such as biocontrol and biofuel production.

## Supporting information

S1 Fig Whole-genome maximum parsimony phylogeny of *Burkholderia ubonensis* relative to other *Burkholderia* species based on 296,578 biallelic SNPs. *B*. *ubonensis* Clades I-VI are labelled, and *B*. *ubonensis* strains from regions other than Australia are noted. Consistency index = 0.36. The tree was rooted with the *B*. *pseudomallei* complex species. In total, 277 taxa were used to reconstruct this phylogeny, of which 264 were *B*. *ubonensis*.
(PDF)

S1 Table Summary of *Burkholderia ubonensis* isolates, sampling sites, accession numbers, genotyping and genomic information, and meropenem Etest results. (XLSX)

S2 Table List of genes and their products encoded by MSMB0022 chromosome III. (XLSX)
