# Supplementary figures and images for: Phylogeographic, genomic, and meropenem susceptibility analysis of Burkholderia ubonensis
Source: PLoS Negl Trop Dis. 2017 Sep 14;11(9):e0005928. doi: 10.1371/journal.pntd.0005928 (PMC5614643; doi:10.1371/journal.pntd.0005928)

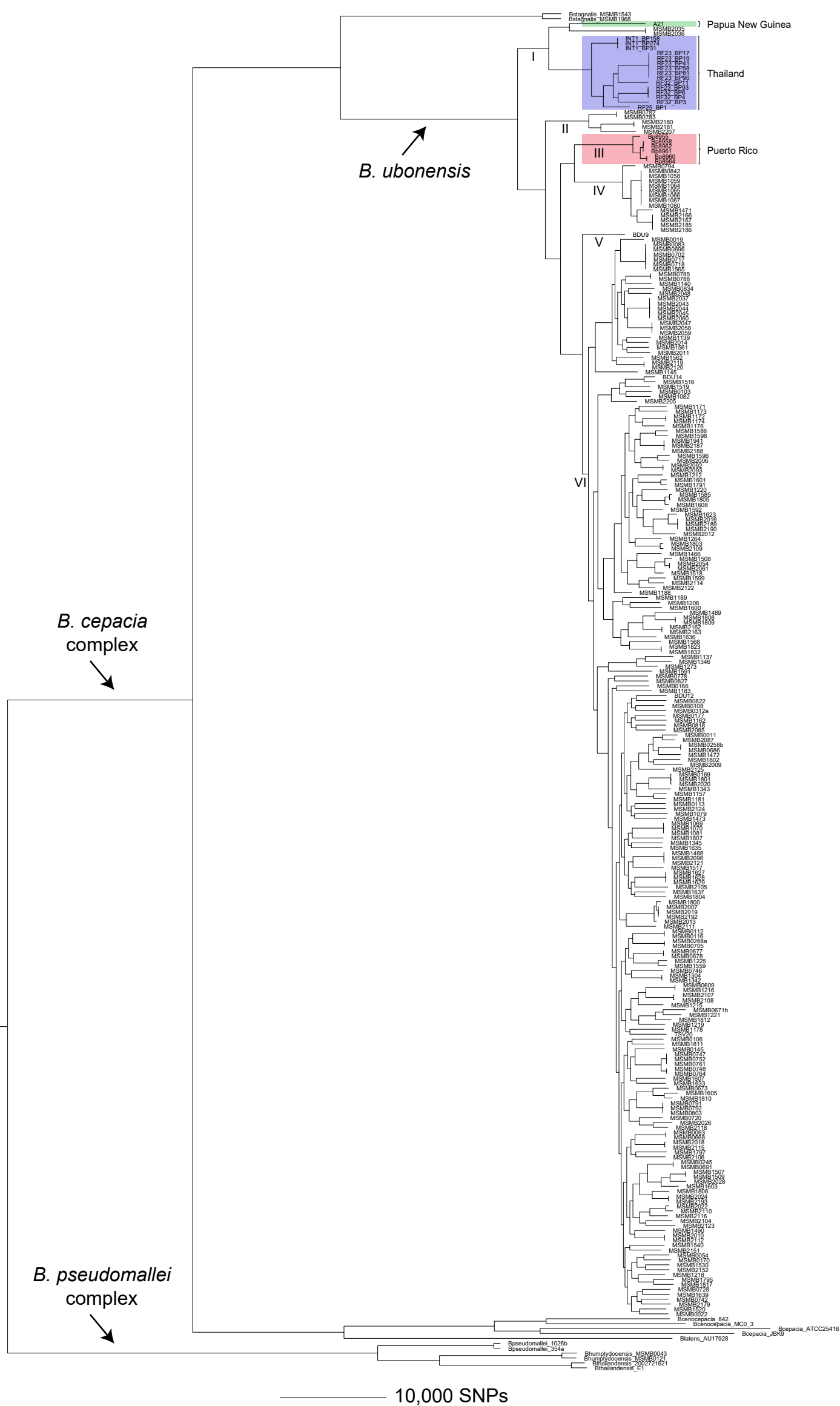

Supplement: S1 Fig — B. ubonensis Clades I-VI are labelled, and B. ubonensis strains from regions other than Australia are noted. Consistency index = 0.36. The tree was rooted with the B. pseudomallei complex species. In total, 277 taxa were used to reconstruct this phylogeny, of which 264 were B. ubonensis. (PDF) [file pntd.0005928.s001.pdf]
